# Detecting Malicious False Frame Injection Attacks on Surveillance Systems at the Edge Using Electrical Network Frequency Signals

**DOI:** 10.3390/s19112424

**Published:** 2019-05-28

**Authors:** Deeraj Nagothu, Yu Chen, Erik Blasch, Alexander Aved, Sencun Zhu

**Affiliations:** 1 Department of Electrical and Computer Engineering, Binghamton University, Binghamton, NY 13902, USA; dnagoth1@binghamton.edu; 2 The U.S. Air Force Research Laboratory, Rome, NY 13441, USA; erik.blasch.1@us.af.mil (E.B.); alexander.aved@us.af.mil (A.A.); 3 Department of Computer Science and Engineering, Penn State University, University Park, PA 16802, USA; sxz16@psu.edu

**Keywords:** video surveillance, visual layer attack, *Electrical Network Frequency* (ENF) signal, False Frame Injection (FFI) attack

## Abstract

Over the past few years, the importance of video surveillance in securing national critical infrastructure has significantly increased, with applications including the detection of failures and anomalies. Accompanied by the proliferation of video is the increasing number of attacks against surveillance systems. Among the attacks, False Frame Injection (FFI) attacks that replay video frames from a previous recording to mask the live feed has the highest impact. While many attempts have been made to detect FFI frames using features from the video feeds, video analysis is computationally too intensive to be deployed on-site for real-time false frame detection. In this paper, we investigated the feasibility of FFI attacks on compromised surveillance systems at the edge and propose an effective technique to detect the injected false video and audio frames by monitoring the surveillance feed using the embedded *Electrical Network Frequency* (ENF) signals. An ENF operates at a nominal frequency of 60 Hz/50 Hz based on its geographical location and maintains a stable value across the entire power grid interconnection with minor fluctuations. For surveillance system video/audio recordings connected to the power grid, the ENF signals are embedded. The time-varying nature of the ENF component was used as a forensic application for authenticating the surveillance feed. The paper highlights the ENF signal collection from a power grid creating a reference database and ENF extraction from the recordings using conventional short-time Fourier Transform and spectrum detection for robust ENF signal analysis in the presence of noise and interference caused in different harmonics. The experimental results demonstrated the effectiveness of ENF signal detection and/or abnormalities for FFI attacks.

## 1. Introduction

Physical infrastructure security and human safety rely on surveillance systems to monitor activities with minimal human intervention. A common example is audio-video systems for detecting human trespassing [1]. Some methods also provide safety by alerting first responders with emergent events to improve safety [2,3]. On the other hand, the proliferation of smart surveillance systems has made them attractive to physical-layer, network-based visual data attacks [4]. These attacks are primarily designed to compromise of audio-video feed to disguise malicious activities or prevent detection. Among them, visual data attacks are a special dimension that only exists in video surveillance systems [5].

*Frame duplication attacks* is a type of visual data attack. It pre-records idle events and upon triggering, replays the pre-recorded video and audio frames to mask current events. Frame duplication attacks result in compromised alarms, which are solely dependent on the surveillance feed received. Even with human intervention to monitor the surveillance data, the malicious activity can go unnoticed. An attacker’s actions could be catastrophic in case of government or banking infrastructure break-ins, where physical security has extremely high priority. Many algorithms have been proposed to detect frame duplication or mirroring attacks [6,7,8], but most of these detection techniques are performed on previously stored media files that can be delayed from event occurrence.

With the proliferation of edge computing and the Internet of Things (IoT) technology, Smart Cities envision public safety surveillance as an edge service. The capability of instant, on-site detection of visual layer attacks, i.e., false frame injection attacks (FFI), becomes essential to keep cities and communities safe [9]. Sensor pattern noise has been used for detecting forgery involving cropping, object removal, and injecting media from different camera sources [10]. However, this spatial forgery detection is not efficient in detecting real-time frame injection attacks where the recorded frames are copied from the same source.

The *Electrical Network Frequency* (ENF) is an instantaneous frequency in power distribution networks, which varies across its nominal frequency 50/60 Hz based on the power supply demand from the consumers. The fluctuation in ENF is typically very close to the nominal frequency [11]. The deviation of ENF from its nominal frequency in the United States is between −0.02, 0.02, whereas in Asian and European countries the fluctuation varies in the order of −0.05, 0.03 from its nominal value [12].The instantaneous behavior of the ENF is useful because the fluctuations are the same within a power grid. The instantaneous values of varying power supply frequency across the nominal frequency are represented as the ENF signal. It has been observed that the surveillance feed contains traces of ENF in both audio and video recordings. The source of ENF in video recording is a light source, like a fluorescent lamp, and in case of audio recording, it could be either from the electromagnetic field interference, mechanical vibrations of electrical powered devices, or the audible hum from powered devices [11].

In this paper, we propose an online authentication system using ENF signal to quickly detect the malicious false frame injection attacks (also referred to as *frame duplication attacks* or *replay attacks*). Specifically, our work is focused on the ENF signal extracted from the audio recordings from the surveillance feed due to its high reliability and efficiency in comparison to video recordings that need a powered light source [13]. The embedded ENF traces are extracted using signal processing techniques like STFT (Short Time Fourier transform), which exploit the presence of ENF signals in multiple harmonics [14]. To establish the extracted signal reliability, the ENF signal is collected directly from the power supply and stored as a reference database. The database includes ENF signal variation w.r.t time and zone of extraction. The major contributions of this work are:

- The feasibility of frame duplication attacks at the edge has been investigated and an attack with smart adaptability to environment and automatic triggering mechanism is implemented and tested;
- The authenticity of ENF signals is validated using signal traces collected at multiple locations within the same power grid;
- A robust method is proposed to extract the fluctuations in audio recordings and to compare with the reference ENF power signal using the cross-correlation factor;
- The relationships between the strength of the acoustic mains hum and the signal to noise ratio (SNR) of the ENF signal are verified;
- The effectiveness and correctness of the proposed detection scheme are validated through an experimental study using real-world ENF signal traces.

The rest of the paper is organized as follows. Section 2 provides the background knowledge of ENF and the related work regarding the attacks on a surveillance system. Section 3 illustrates the feasibility of launching a frame duplication attack at the edge through actual implementation. Section 4 introduces our method to detect false frame injection attacks utilizing the ENF signals embedded in the recorded audio and provides available techniques on video recordings. Section 5 presents the experimental results that verify the effectiveness of the proposed method. Section 6 concludes this paper along with a brief discussion regarding our future work.

## 2. Background Knowledge and Related Work

### 2.1. Attacks on a Surveillance System

Nowadays, video surveillance systems are arguably the most popular measure for the safety and security of physical facilities and residents of communities. The emergence of more sophisticated attack tools and methods has brought deep concerns to researchers and stakeholders. Network-based attacks like cross-site scripting, buffer overflow, SQL injection, and boot loader or firmware attacks give privileged access to unauthorized people. Gaining root access allows attackers to impair the normal function of a surveillance system by conducting more attacks, such as blinding cameras, disabling video sensors, eavesdropping, as well as data exfiltration and infiltration oriented visual-data layer attacks. These suspicious activities could escape from detection and the attacker may even gain command and control over the surveillance network [5].

In this paper, we focus on *data infiltration based visual-data layer attacks*. Frame duplication attacks are one of the most frequently encountered forgery attacks on a live video feed. Once the attacker has gained access over the surveillance cameras through network attacks, the attack code can control the surveillance output. By inserting previously recorded video and audio frames with normal scenarios, the on-going suspicious activities, personnel, or objects may go undetected. Many methods have been proposed to detect the replay attacks using spatial and temporal domain similarities by extracting features from the video frames and analyzing these features to detect frame forgery [15]. These algorithms mostly extract features from a video sub-sequence and compare them with other sub-sequences for similarity [16]. A number of correlation techniques [6,17,18] have also been adopted to identify frame duplication and region duplication in a video. All these similarity detection techniques require a stored surveillance recording database, and hence they require much computation time to process each video frame. In the case of surveillance systems, the late discovery of such forgery after the event does not afford intervention, incident capture, or property anti-theft. Real-time detection and alarm indication is a top priority.

In order to launch a false frame duplication attack, the attack code works in a controlled environment. It is recognized that environmental factors change continuously, like the light intensity of the surroundings due to daytime or nighttime, an object’s position in the point of view (POV) of the camera, or the introduction of new objects [9]. If there are visible differences between the pre-recorded frames used for attack and the current genuine frames, the security personnel may beware of it immediately. Hence the attack code continually looks for any change in the camera’s POV and updates the pre-recorded frames with the new changes made in the environment. The environment monitoring allows the attacker to always have up-to-date recorded frames which can be triggered at any instant. For example, using simple facial recognition software in the attack code, an attacker can launch the attack upon detecting a specific face (or as simple as a quick response (QR) code). In this paper, for demonstration we will use a face detection based trigger to launch the attack, and collect the surveillance feed for analysis (discussed further in Section 3).

### 2.2. Electrical Network Frequency Signals

ENF signals can be extracted using various techniques from both audio and video recordings. The collection of ENF signals is also affected by many factors including the environment of recording and the recording device itself. Initially, ENF traces were found in recorders that were directly connected to a power grid, and other researchers showed that ENF signals are also present in battery-powered devices [19,20]. The source of ENF in such battery-powered devices is the audible hum from any electrical device running on power from the main grid and generating noise, where the noise carries the time-varying nature of ENF traces [20,21]. For battery-powered devices, a device in motion can have high noise and interference caused due to air friction in the ENF frequency zone, hence making ENF extraction more difficult [22].

In this paper, ENF signals are extracted from audio recordings made by surveillance cameras connected to the power grid. Audio signals are recorded at a sampling rate of 8 KHz. This sampling rate provides room to capture the ENF traces in multiple harmonics including the nominal frequency of 50/60 Hz and consumes less storage. Meanwhile, the high video frame rate of surveillance cameras makes it difficult to capture the ENF that varies with high time resolution. Some earlier research has extracted the ENF signal by capturing changes in light intensity using optical sensors, aliasing frequency, rolling shutter, and a super-Pixel based approach [13,23,24]. However, these techniques are computation intensive, which makes them impractical for edge devices.

**Figure 1:**
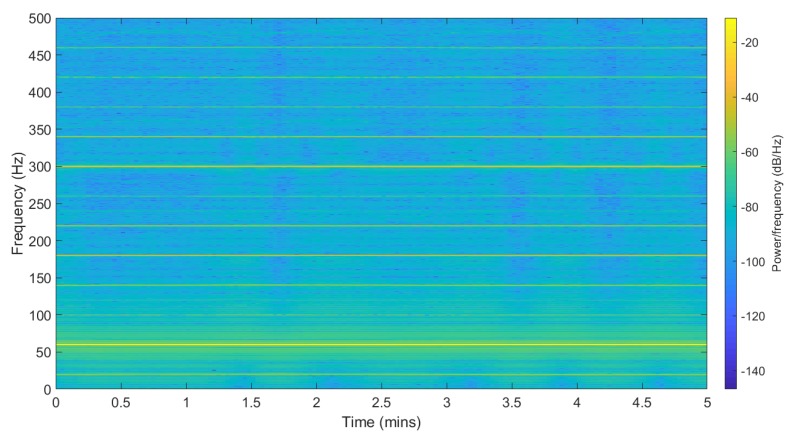
Spectrogram of power recording.

ENF signals can be collected using a circuit consisting of a step-down transformer and a voltage divider circuit. Figure 1 shows a spectrogram of the collected power recordings with the ENF traces embedded in it. The SNR ratio is high around the nominal frequency zone. The example is recorded at Binghamton University in the United States, so the nominal ENF frequency is around 60 Hz and it varies in the range of ±0.02 Hz. The range of variation changes per the location; for instance, India and Lebanon have a frequency variation around the nominal frequency in the range ±0.8 Hz. The ENF variations are observed to appear in many harmonic bands along with the nominal frequency band [14]. These harmonic bins have different signal strengths as compared to the nominal bin.

**Figure 2:**
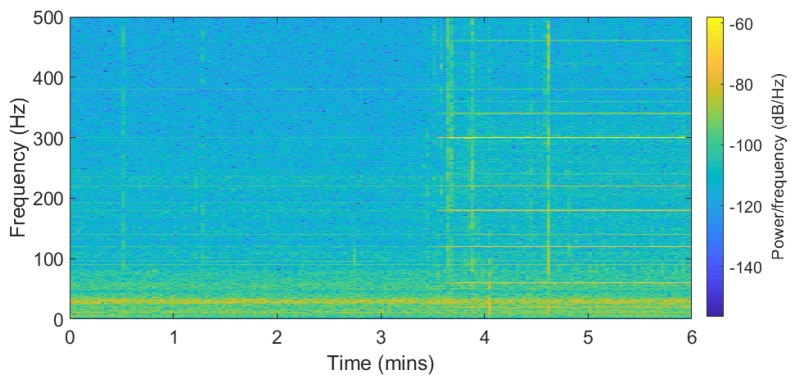
Spectrogram of audio recording with noise source after 3.5 min.

Figure 2 represents the audio recording spectrogram. The recording was made in an android phone connected to the power supply for six minutes, where the first 3.5 min were recorded without main power electrical devices like computers or speakers operating nearby. Then, after 3.5 min the electrical devices around the recorder were powered on. The ENF traces are available in the nominal frequency along with its harmonics for the second part of the recording. In the first part of the recording, the ENF traces were captured as a result of direct power grid connection. It is also possible that the traces were captured due to low-energy ambient noise from devices running farther away from the recorder. The recordings show that ENF traces can be captured in the presence of acoustic hum or from devices directly connected to the power grid.

### 2.3. ENF Signal Applications

ENF signals have been adopted in digital forensics to authenticate digital media recordings [11,21]. The use of the ENF technique was first demonstrated to authenticate media recordings as proof for legal jurisdiction purposes so as to verify whether or not evidence was tampered with. The ENF authentication technique was introduced and multiple extraction processes have been discussed [11]. Many forgeries as false evidences were detected using the instantaneous ENF signal. Robust extraction of the ENF signals has been an active research topic and multiple signal extraction and tracking algorithms have been proposed [11,25,26,27]. The signal extraction experiments on alternating-current (AC) powered recording devices and battery-powered devices reveal the source of ENF in a battery-powered device is the acoustic hum generated by the electrical devices connected to a main power source [20,21]. These experiments show that the main power noise source in the proximity of the recording devices can result in capturing ENF traces.

A high precision phase analysis technique was introduced, which checks for sudden changes in the phase and amplitude of the extracted ENF signal [27]. This technique does not rely on a pre-built reference database, but there were cases where the deleted or added video clip could have the same phase as the proceeding clip. Hence, there are no observable phase or amplitude changes to utilize. As the ENF signals are embedded in multiple harmonics along with the nominal frequency range, a multi-estimator model could enable a more robust extraction of ENF signals from a weak spectral component [14,25]. The estimator model states that the frequency variations of the harmonic spectral range have a larger variance when compared to the nominal frequency. It has also been observed that for different types of recording environments, recording devices with different microphones like dynamic or electric microphones result in ENF traces with high SNR in specific harmonic ranges as compared to the rest of the spectra. The extraction process includes combining multiple spectral frequency ranges, resulting in a robust signal with a low computational requirement.

**Figure 3:**
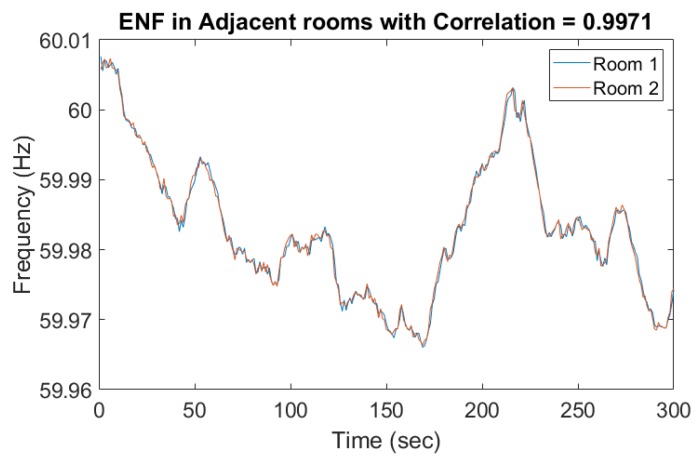
Electrical Network Frequency (ENF) captured in adjacent rooms.

**Figure 4:**
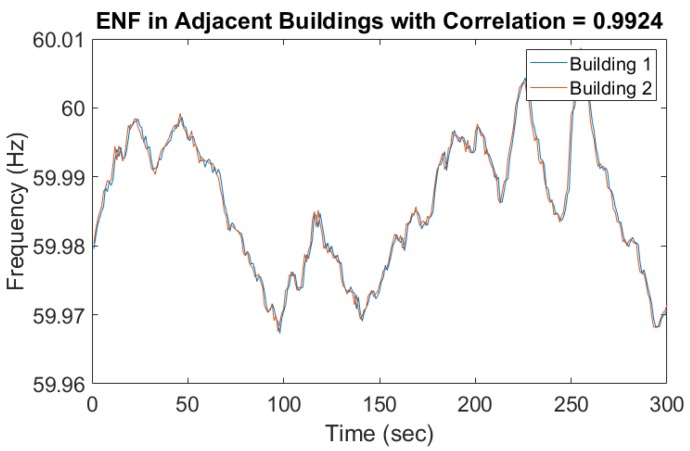
ENF captured in adjacent Buildings.

These previous studies demonstrate the usefulness of ENF, so we adopted this technique to extract ENF signals from our surveillance recordings. Various environmental factors and device-related scenarios, like wave interference, the Doppler effect, and movement of the recording device with respect to the noise source could affect ENF capture in the audio [22]. For instance, due to the different types of microphones used, the ENF signals were embedded in multiple harmonics. Figure 3 and Figure 4 represent two ENF instances recorded at the same time in two different rooms and different buildings. The ENF signals were very similar throughout a power grid and the slight shift might be due to the oscillator error in two different device recorders.

Algorithms to extract the ENF signals from video recordings along with the audio samples can be developed simultaneously. For example, ENF traces can be detected in video recordings using optical sensor measurements with indoor lighting [13]. A light source was required during video recordings. Additionally, the availability of ENF traces in surveillance camera video recordings made using average pixel intensities per frame was confirmed using frequency aliasing. An alternative approach to extract ENF fluctuations from CMOS camera recordings uses rows from each video frame leveraging a rolling shutter technique [28]. This technique cannot be universally applied to all cameras since the idle period at the end of each frame varies per camera manufacturer. Although for pre-determined surveillance cameras, the idle period can be estimated beforehand and improve ENF sampling frequency.A Super-Pixel based approach divides a video frame into a group of pixels with similar pixel intensity as known as *super-pixels* [24]. The instantaneous light condition variations in these super-pixels are used to detect the presence of ENF in a given video file without investing a lot of processing power and time on video files with no ENF traces.

ENF is also used as a source for multimedia synchronization, where normalized correlation coefficient estimates the lag between peak correlation values which determines the shift required for synchronization. An absolute error map obtained between ENF signals from a reference database and estimated from media recordings allows tampering detection and timestamp verification [29,30]. The error map technique requires computing the absolute error map for every index and shift of signal along with a line detection algorithm with an exhaustive point search and measurement. An ENF error map algorithm benefits in a situation where the media files are pre-recorded and reasonable computing resources are available.

## 3. Real-Time Frame Duplication Attack Implementation

Before introducing our ENF-based detection mechanism, this section investigates the feasibility of an automated real-time frame duplication attack at the network edge by an experimental case study. The constructed attacking system also serves as the testbed for detection scheme validation.

### 3.1. Overview

To launch a real-time frame duplication attack, we assume that the edge based surveillance systems have been compromised through network attacks. This allows the attacker to gain complete access to the live video feed along with the manipulation of the output stream as required. The algorithm devised includes two modules, monitoring for audio-video replay and deploying an attack.

**Figure 5:**
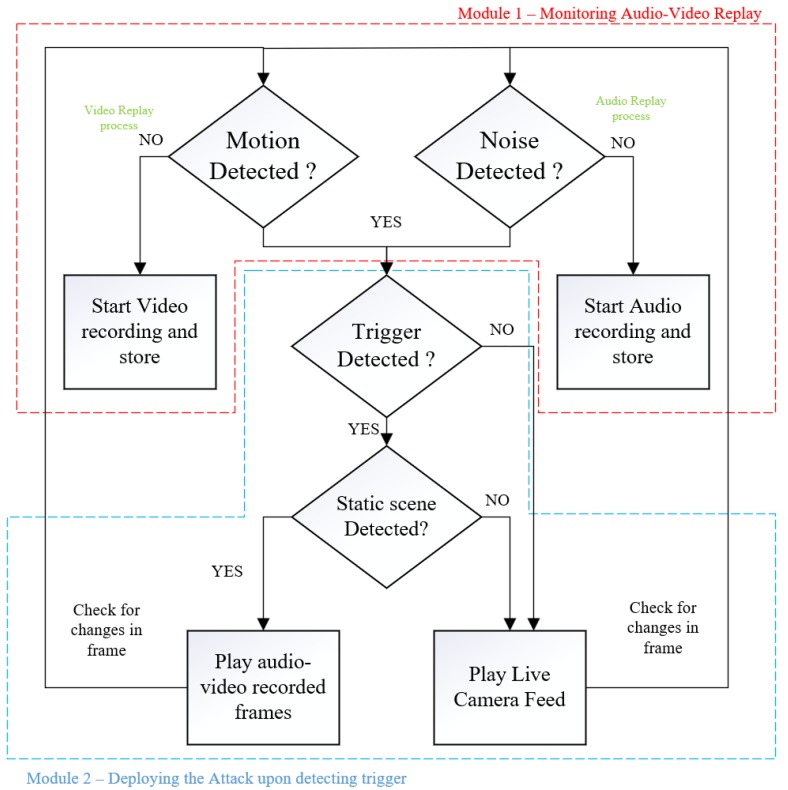
Flow diagram of the frame duplication algorithm.

Figure 5 represents the algorithm flow diagram. In the first module “monitoring audio-video replay” consists of collecting a duplicate recording in two parallel processes where video and audio streams are monitored independently. The video monitoring process constantly checks for any motion in the frame and when a static scene is detected, an automated recording of the static scene is started in the background process. The motion detection algorithm in the video process performs a Gaussian blur on the frames to smooth out the edges and minimize errors due to noise, and then changes in pixel intensities are compared with a threshold to detect any motion. The audio monitoring process detects noise in the environment and records audio samples when there is no background noise. With the “monitoring audio-video replay”, a recent recording of the video and audio are collected and stored. The second module “deploying an attack” represents detecting a trigger and launching the attack. The mechanism used as a cue is the face recognition algorithm. When the trigger event is detected, the video frames and audio samples are combined and deployed to mask the live video feed.

### 3.2. Attack Algorithm Functionality

The monitoring audio-video replay module discussed earlier consists of two parallel processes, for video and audio, running independently to collect replay recording. The term *replay recording* represents a pre-recorded video frames or audio samples to be used later by the algorithm when the attack is triggered. The motion detection algorithm in the video process is used to detect an occurrence of a static scene by comparing the pixels in consecutive frames. The changes in pixel intensity are compared with a threshold, where different environments have a different sensitivity to pixel changes and hence different threshold values.

For our testbed, we considered indoor environments where the changes in pixel values were more stable in comparison to outdoor environments. The changes which occur indoors are people walking, gradual changes in natural light intensities, and artificial light changes. The algorithm was tuned to detect these changes in the frames by using a Gaussian blur on incoming frames. The Gaussian blur performs convolution on the image, acting as a low pass filter and therefore attenuating high-frequency components more than the lower-frequency components. Since human movement in the camera view appears as a low-frequency change while noise is a high-frequency change, removing the noise helps the algorithm better distinguish human motions from noise. Below is the Gaussian function for calculating the transformation to apply to each pixel in the image:

$$G\left(x,y\right)=\frac{1}{2\pi \sigma^{2}}e^{-\frac{x^{2}+y^{2}}{2\sigma^{2}}}$$

where $\sigma^{2}$ is the variance of the Gaussian distribution, and *x* and *y* are the distances from the origin in the horizontal axis and the vertical axis, respectively.

In visual replay attacks, a duplicated streaming video out of synchronization with its audio could potentially raise suspicions to people monitoring the surveillance. Hence, the second parallel process, where the audio process is running to detect static noise in the environment and collect audio replay recordings. For example, if a static video is replayed in the live feed and the audio in the background has surrounding noise which is independent of video, it would raise suspicions. So, the video frames and audio samples are recorded independently and replayed together to represent a static scene with no background noise. The audio replay recording is collected when there is no noise detected. A Fast Fourier Transform (FFT) is performed on the samples to obtain a frequency domain representation of the input audio stream. Noise detection is performed by taking the mean volume across all frequencies and comparing it to a threshold. The threshold for audio is also decided based on different environmental settings of the camera.

The trigger detection in the “deploying attack module” is responsible for detecting a pre-determined event and using the audio-video replay recording as pseudo-live feed. In this paper, face detection (of the attacker) module was used as a triggering event. For modern surveillance cameras, a high-quality video stream was captured with decent frames per second (FPS) compared to the surveillance cameras a decade ago. For the face detection module, the FPS processed was lower, but a single frame with the required face model detected was enough to trigger the attack and make the processing speed irrelevant. In the face detection module, we used histogram of oriented gradients (HOG) for fast human/face detection [31,32]. The gradients of human faces were trained using a machine learning algorithm, where each face has a unique encoding. The perpetrator’s face encoding was generated beforehand and embedded in the algorithm. When the perpetrator showed up in the camera view, the encoding vector was detected, and this event was used as a trigger mechanism for the replay attack. To avoid suspicions by deploying the attack as soon as the face was detected, the attack was instead placed on hold until a static scene appeared again, and then the frames were replayed to mask the live feed.

The face detection model was used as an example to demonstrate the remote triggering capabilities of malicious algorithms. The trigger mechanism could also be performed manually using a command and control server to communicate with all the compromised surveillance cameras or by using a naturally occurring event to leave no traces of the attacker appearing in the frames. Other examples of a triggering event could be a specially designed QR-code on a T-shirt, a unique hand gesture, or even a voice-activated trigger.

**Figure 6:**
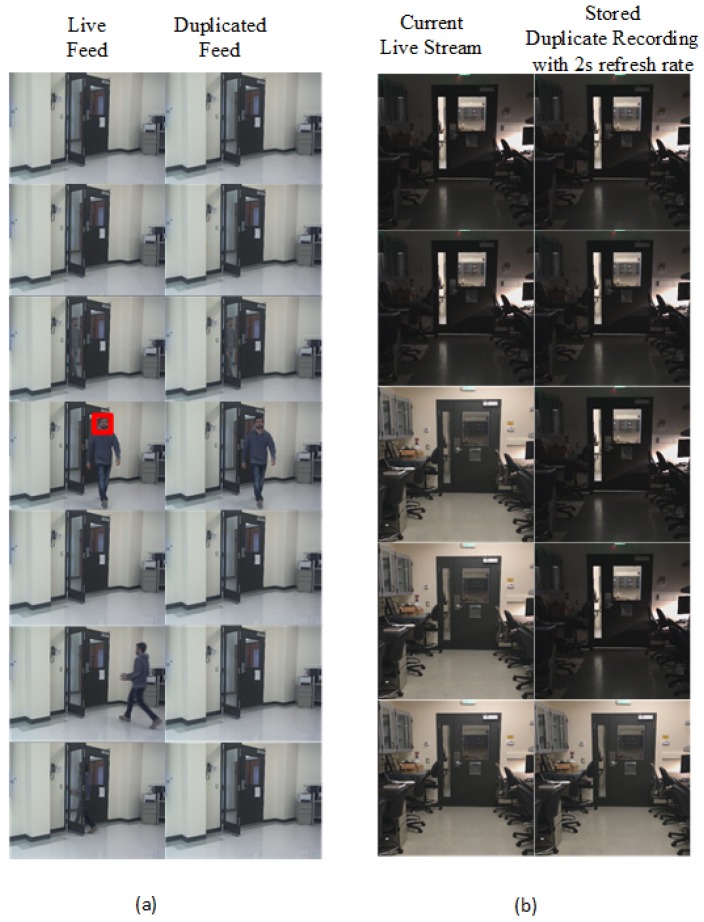
Frames of live and duplicated feed. (**a**) Attack triggered after detecting a face encoding and launched when a static scene appears, and (**b**) updating the duplicated static scene recording based on changes in light intensity of the environment.

Figure 6 shows the frames observed by the camera (i.e., “live feed”) and frames captured or delivered by the camera (i.e., “duplicated feed”) when the attack is launched. In Figure 6a, the face encoding of a user (i.e., perpetrator) has been stored in the algorithm. When the perpetrator enters the scene, the camera detects the face along with other faces in the scenario. The perpetrator could walk into the scene with a group or individually, as long as the camera can detect the face and match it with the embedded face encoding. The HOG encoding is unique for different face structures, and hence it is faster to deploy a facial recognition algorithm at the edge. Once a static scene is detected, the duplicated frames are replayed. Here, we opted to deploy the attack once a static scene appeared again instead of immediately launching the attack. Deploying the attack with static scene avoids suspicious artifacts like the sudden disappearance of a person from frame, and detecting duplicated frames in a static scene is harder than frames with objects in motion [18]. In Figure 6b, the periodic changes in the environment is reflected in the replay recording. The algorithm checks for changes in the environment every two s and updates the stored recording accordingly. The second column represents the recording stored for future deployment of attack. The duration of the recording made is also modified based on the indoor-outdoor requirements. The capability of the attack algorithm to adapt to changes in real-time shows the reliability of the algorithm in fooling human perception and reducing suspicious behavior when a camera does not reflect the changes according to the environment. For example, a replay recording made at noon is used at night time; this can easily raise suspicion and alarm the authorities.

**Figure 7:**
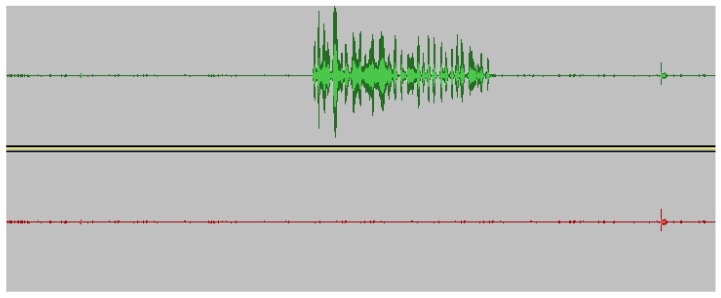
Original audio masked by replay recording of noiseless background recording. The top recording represents the original audio recording and the bottom recording is the duplicated recording after the attack is launched.

Along with the video frame duplication, the audio samples are also masked. Figure 7 represents masking noise made during the replay attack with its pre-recorded audio samples with no or less background noise. The allowed noise depends on the threshold used to compare the frequencies in FFT. For an indoor application, the noise level is assumed to be minimal, so higher frequency noise is eliminated from the replay recordings.

## 4. Detecting Malicious Frame Injection Attacks Using ENF Signals

Inspired by the characteristics of ENF signals, this work explores the feasibility of applying it to detect malicious frame injection attacks at the edge. In order to obtain a reliable ENF signal from the surveillance systems, we opted to use audio records as the source, which is insensitive to light conditions. A reliable database for authenticating the extracted ENF was created utilizing robust extraction techniques like the spectral combination of multiple harmonics. A *correlation coefficient threshold based* method was introduced to detect the existence of duplicated frames inserted by the attacker.

### 4.1. Applied Model

ENF traces occur around the nominal frequency range 50/60 Hz as $f_{ENF}=f_{o}+f_{\Delta }$, where $f_{o}$ is the nominal frequency and $f_{\Delta }$ is the instantaneous frequency fluctuations from the nominal value. For power recordings, Figure 1 shows the ENF traces at odd multiples of harmonic, with a strong signal at 60 Hz. In case of audio recording, Figure 2 shows that the traces occur more around even harmonics depending upon the type of microphone used.

For the spectrogram calculation of the recorded signal, we used a frame size of 1 s and nFFT = 8192, which gives a frequency resolution of 0.122 Hz for a signal with a sampling rate of 1000 Hz. The length of recorded signal used for each instance is 6 s. The power spectral density (PSD) of the ENF carrying signal is used to extract certain spectral bands s(f), where the PSD S(ω) is computed from the FFT of the signal and $f\in k[f_{o}-f_{v},f_{o}+f_{v}]$. $f_{v}$ is the variation width of the ENF signal, $f_{o}$ is the nominal frequency, and *k* represents the harmonic frequency band.

The PSD $S_{N_{XX}}\left(f\right)$ is:

$$S_{N_{XX}}\left(f\right)=\frac{1}{N}{\left|X_{N}\left(f\right)\right|}^{2}$$

where $X_{N}\left(f\right)$ is the Fourier transform of the signal:

$$X_{N}\left(f\right)=\sum_{n=-\infty }^{\infty }x_{n}e^{-j\omega nT}$$

where w=2πf, *T* is the period of the signal duration, and *n* is the number of samples 1≤n≤N. Sampling at discrete times $x_{n}=x\left(n\Delta t\right)$ for a period T=NΔt, the PSD is:

$${\bar{S}}_{XX}\left(\omega \right)=\frac{{\left(\Delta t\right)}^{2}}{T}{\left|\sum_{n=1}^{N}x_{n}e^{-j\omega n\Delta t}\right|}^{2}$$

From the obtained spectral band, the instantaneous frequency for each frame window used is estimated by the maximum value in each power density vector obtained for that time instant. The period of signal duration represents the number of vectors obtained from PSD and instantaneous ENF values. Quadratic interpolation is used to obtain its dominant frequency from the maximum value in each vector. In quadratic interpolation of the spectral peak, the peak location is given as:

$$\Delta =\frac{1}{2}*\frac{\alpha -\gamma }{\alpha -2*\beta +\gamma }$$

where α is the previous bin of the max spectral bin, β is the max spectral peak and γ is the next bin. If $k^{*}$ is the bin number of the largest spectral sample at the peak, where $1\leq k^{*}\leq K$ for K bins, then $k^{*}+\Delta$ is the interpolated peak location of the bins and the final interpolated frequency estimate is

$$f_{\Delta }=\left(k^{*}+\Delta \right)\frac{f_{s}}{N}$$

here $f_{s}$ is the sampling frequency and *N* is the number of FFT bins used. The instantaneous frequency estimate of the ENF signal is then given as $f_{ENF}=f_{o}+f_{\Delta }$.

### 4.2. Robust Extraction of ENF signals

**Figure 8:**
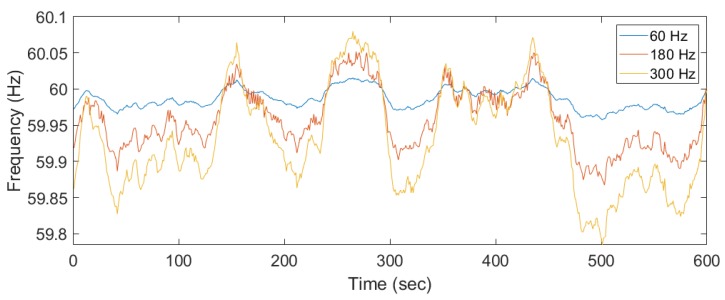
Different harmonics of power recording shifted to 60 Hz for comparison.

ENF traces appear in different harmonics with increasing frequency variations at different spectral bands. Figure 8 shows similar ENF fluctuations at odd/even harmonics. The power recordings were not affected by any noise since it was directly extracted from the power outlet, but in case of audio recordings, external noise could be captured and interfere with the ENF frequency ranges. The noise could lead to an inaccurate estimate of the ENF signal. A more robust technique was proposed to combine the spectral frequency bins from different harmonic bins based on the SNR [14]. The SNR is represented as the weight of spectral band, computed as the ratio of the mean of the PSD in the ENF frequency range to the mean of spectral bin of that harmonic frequency.

$$w_{k}=\frac{\sum_{k=1}^{L}s\left(f_{o}-f_{c},f_{o}+f_{c}\right)}{\sum_{k=1}^{L}s\left(f_{o}+f_{c},f_{o}+f_{v}\right)+s\left(f_{o}-f_{c},f_{o}-f_{v}\right)}$$

where $f_{c}$ is the range of ENF variations, and it is typically 0.02 Hz in US and varies in European and Asian countries. $f_{v}$ is the spectral band of interest in each of the *k* harmonics and $f_{o}$ is nominal frequency. The weight obtained from each spectral bin is normalized and combined with different spectral bins to compute a combined spectrum of all harmonics containing ENF.

$$S\left(f\right)=\sum_{k=1}^{L}w_{k}s\left(f\right)$$

The normalized weight represents the SNR of harmonic frequency in different bands. The noise in some frequency band can be eliminated for the spectral bands with very low SNR. The approach is computationally more intensive for edge devices, therefore, a fog node was used to perform a second pass on ENF estimation on the audio recordings with more robust extraction by eliminating the false alarms produced by the edge devices. The discussion of the edge-fog-cloud hierarchy is beyond the scope of this paper—interested readers may find the architecture description in our related publications [33,34,35]. Along with robust audio-based ENF extraction, video-based ENF extraction module could be added. The video module includes processing video frames with moving subjects, which requires higher computational power for computing super-pixels, averaging pixels per frame or per row, and using alias frequency for ENF estimation. Although this additional processing can be integrated with the fog node with higher availability of computational power and double authentication process. The drawback is that for the major part of the day, the chances of presence of light source is low, hence there is no source of ENF in the video recordings.

### 4.3. Correlation Coefficient for Extracted ENF Signals

The ENF signal estimated from both the power recording and audio recording for a small duration were compared to check for similarity using a correlation coefficient between the two signals [36]. The ENF signal from power $P_{ENF}$ and audio $A_{ENF}$ is given as:

$$\rho \left(l\right)=\frac{\sum_{n=1}^{N}\left[f_{P_{ENF}}\left(n\right)-\mu_{P_{ENF}}\right]\left[f_{A_{ENF}}\left(n-l\right)-\mu_{A_{ENF}}\right]}{var\left(P_{ENF}\right)*var\left(A_{ENF}\right)}$$

where $f_{P_{ENF}}$ and $f_{A_{ENF}}$ are the frequency estimation of the ENF signal from power and audio recordings, respectively. μ and var are the mean and variance of the frequency signal. *l* is the lag between the two signals. Even though the recordings are made at the same time, due to the oscillator error between the two devices the signals are not in sync. The lag is used to match the signals and a threshold decides the similarity between the two signals. If the difference between the reference and the current detection goes beyond a certain threshold, the system considers that a false frame injection attack is detected.

## 5. Experimental Results

### 5.1. Testbed Setup

A Raspberry Pi Model B was used as an edge device where the surveillance system was operating. An additional module with a sound card was added to record the power recording at the same time as the audio recordings. A Python based code was used for the implementation and estimation algorithm of the ENF signal. The Python’s parallel threading enables capturing and estimating the power ENF and the audio ENF simultaneously. The recordings were stored as a file in the common database. A laptop was used as a fog node to estimate the same ENF signals to verify the signal correlation in the second pass. Power recordings were made using a step-down transformer and a voltage divider circuit [11] and given as an input through a 3.5 mm audio jack. To reserve the computational power, the recordings were made in mono channel instead of a stereo channel. The signals were recorded at the sampling frequency of 8 K Hz and was down sampled to 1000 Hz for estimating the signals.

### 5.2. Implementation and Results

**Figure 9:**
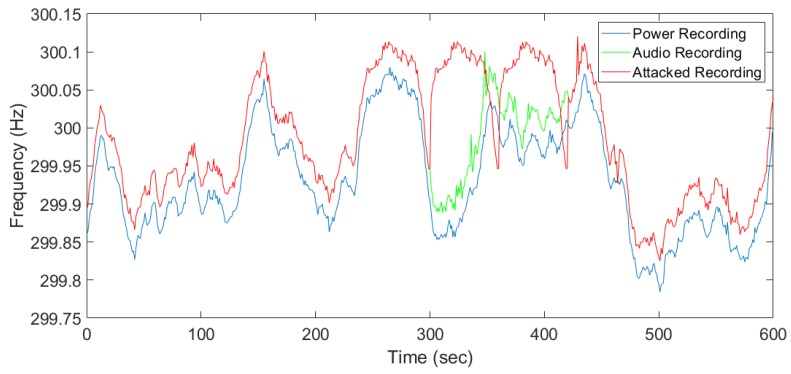
ENF estimated from the power and original audio recording.

Both power and audio recordings with ENF traces were made simultaneously and the estimated ENF signals were compared based on the correlation coefficient obtained. We have implemented a visual-data layer replay attack and collected both the original and attacked audio recording along with the power recording simultaneously. Strong ENF traces were observed at 300 Hz for both power and audio recordings. Figure 9 presents the estimated ENF from the power, original audio, and attacked audio recording, respectively. The attacked audio includes pre-recording a selected period of time and was replayed to mask the current original recording. The attack was launched at 300 s and a clear deviation between the original recording (green signal) and the attacked recording (red signal) can be observed. The part of the recording which was replayed is clearly seen from the signal comparison as the ENF estimates do not match, which indicates the possibility of forgery attacks on previously recorded media files. The correlation coefficient between the power ENF signal and the attacked ENF signal will be lower for the replayed part of the recording. Figure 9 conceptually validates the idea that ENF traces to distinguish an anomaly incurred by the injected frames.

**Figure 10:**
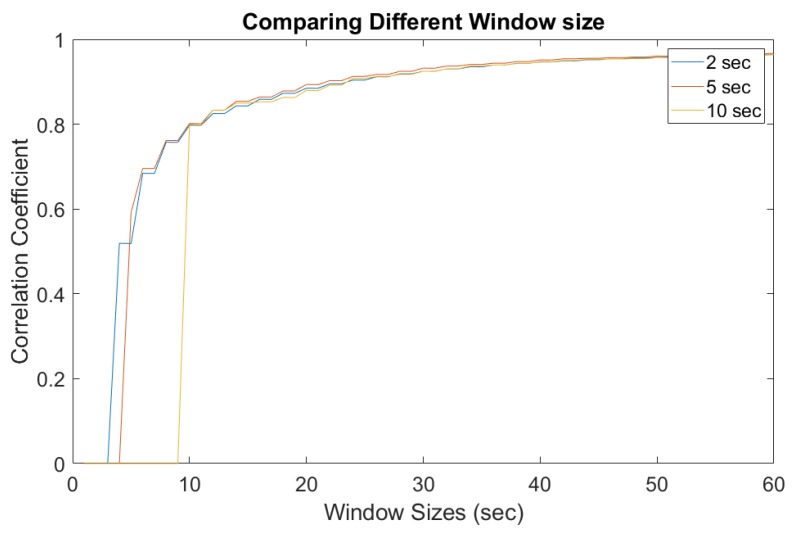
Window sizes for power and original audio.

**Figure 11:**
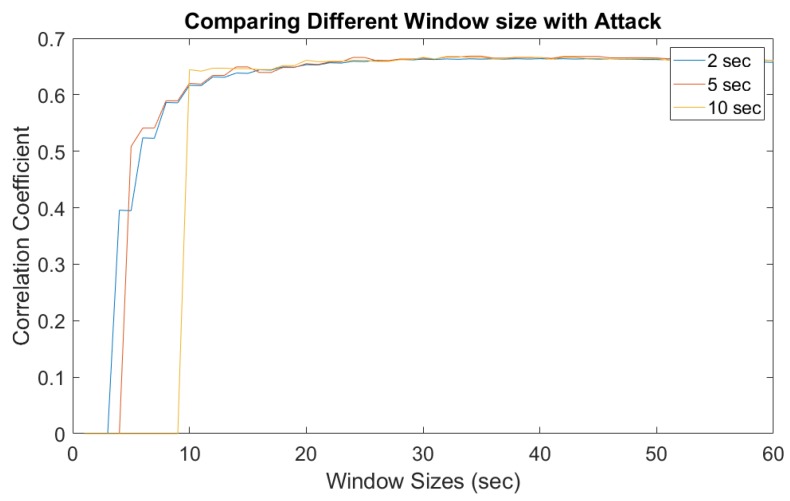
Window sizes for power and attacked audio.

In practice, a responsive surveillance system has to provide alerts instantly rather than help discern the problem from a delayed forensic analysis. Therefore, a sliding window-based approach was introduced to extract and estimate the ENF from online records. A thorough study was conducted for a better understanding of the different setup and overlap times between each ENF estimates. Comparisons were made with the correlation coefficient between those estimated ENF signals. Figure 10 and Figure 11 show different window sizes used at the initial process. Based on the comparison between different shifting step lengths, it is clear that a window size of 25–30 s is the minimum to obtain a constant correlation coefficient of 0.8 and this value can be used as a threshold to detect dissimilar ENF signal estimations. Figure 10 is the correlation coefficient between the power signal and original audio signal. Figure 11 is estimated between the power and attacked audio signal. It is clear that the correlation was higher for the original audio signal compared to the attacked audio.

**Figure 12:**
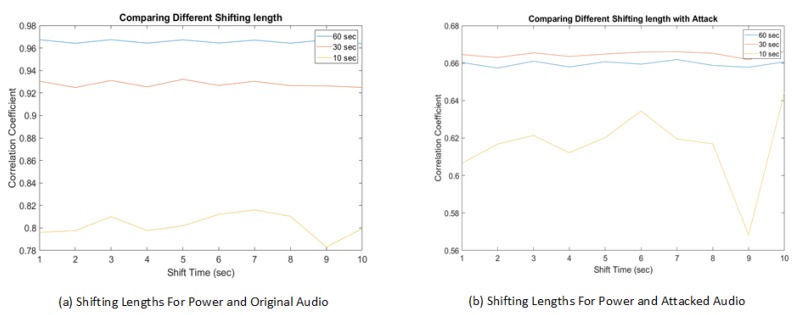
Shifting lengths comparing power with the original and attacked audio.

Figure 12 is a detailed comparison between different window sliding step sizes. It is clear that with a smaller step size, a higher correlation coefficient value was obtained compared to larger step sizes. However, the experimental study also shows that the computational overhead was higher with the smaller sliding window step sizes. A balanced point is that a window step size of 5 s allows a real-time response in case of mismatching signals. Taking multiple factors into consideration, our experimental results suggest a threshold for a correlation coefficient between two signals to be 0.8. A correlation coefficient above the threshold value of 0.8 means the video/audio stream is normal, while below 0.8 implies the possible existence of injected false frames. The lower the value is, the higher probability of attack.

**Figure 13:**
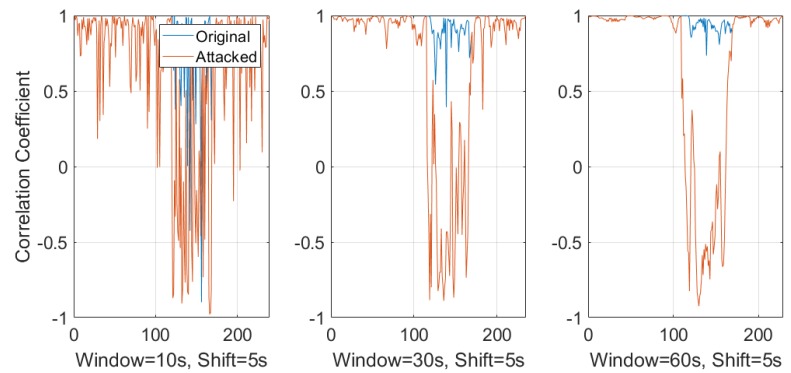
Correlation coefficient with 10 s, 30 s, and 60 s sliding window with a shift step size of 5 s.

**Figure 14:**
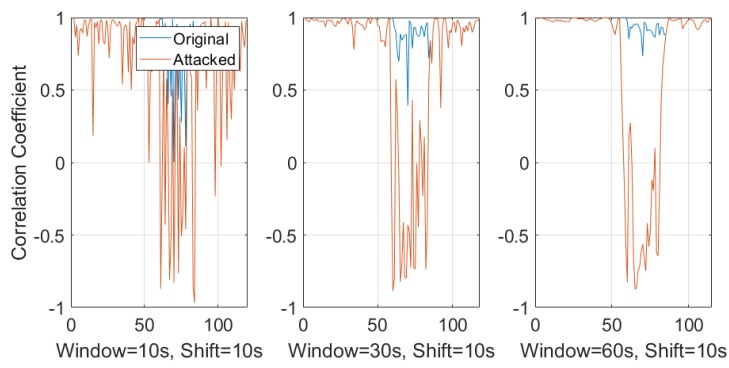
Correlation coefficient with 10 s, 30 s, and 60 s sliding window with a shift step size of 10 s.

Figure 13 is the comparison between different window sizes with a sliding window shift step size of 5 s. Even though the window size of 10 s has smaller initialization delay, it is susceptible to a high false positive rate. The fluctuations in the correlation can be seen for original recording where some windows are not similar. In case of a 30 s or 60 s window size, the detection of frame duplication attack is similar. The 60 s window has less fluctuations between adjacent windows and the threshold of 0.8 clearly separates the distribution of duplication attacking scenarios with the actual normal recording. Comparing with Figure 14, which represents a window shift step size of 10 s, it is clear that the shift step size has a lower impact compared to the window sizes.

### 5.3. A Case Study on Foscam Camera

**Figure 15:**
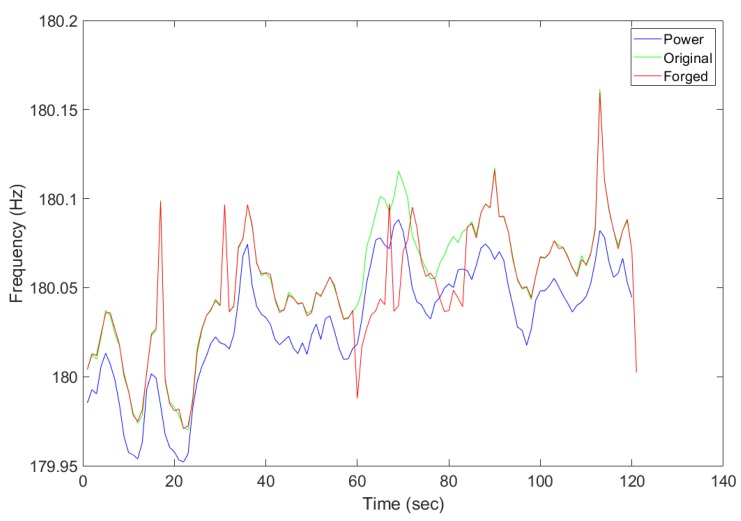
Comparing the recording collected by the attacked, original, and power recordings.

**Figure 16:**
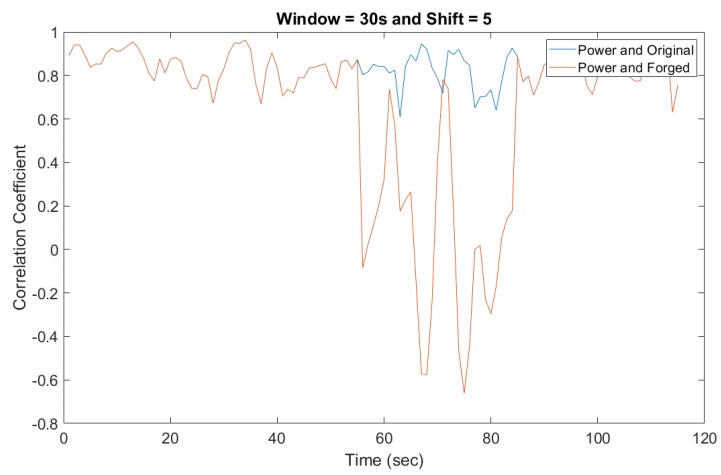
Detecting the forged audio recording using correlation coefficient.

Additionally, the proposed detection method was been implemented and fully validated on a real video surveillance system using a cheaper FOSCAM camera. The Foscam surveillance camera tested was powered directly from the electrical grid. As proof of concept, the Foscam API was used to control the camera recordings through python scripts. The resulting parameters were tested using the forged and original audio recordings. Figure 15 shows the ENF extracted from the audio recording along with parallel power recording. As the estimated ENF shows, while the original audio/video was masked by the forged audio/video, the drop in the correlation coefficient was observed in Figure 16, which clearly indicates the detection of an anomalous activity.

In summary, the collected data and experimental results conclude that it is worthy to have a higher initialization setup delay with a better performance with an average shifting length of 5 s. In order to reduce the false alarm rate, a consecutive lower correlation coefficient detected by the system can be treated as an immediate alert to a challenging situation. In addition, a second pass performed by the fog layer can also be used as a reassurance for the alert.

## 6. Conclusions

Increasing the number of attacks on smart surveillance systems presents more concerns on security. In this paper, we discussed a visual-data layer attack on video surveillance systems and introduced a novel detection method leveraging ENF signals. ENF fluctuations were inferred to be similar at different locations at the same time instantly, and these ENF traces were embedded in media recordings through various factors. The ENF estimations from power and audio recordings were estimated simultaneously, and a correlation coefficient was used to evaluate the signal similarity. A low correlation coefficient indicated that the signals were not similar, which in turn implied the potential existence of maliciously injected duplicated frames. A sliding window-based approach was proposed for online detection and different parameter values were investigated to obtain the best setting.

While the proposed system was focused on audio recording to detect frame duplication attacks using ENF fluctuations at edge devices at a low computational cost, it was also possible that the ENF harmonics were contaminated due to other electromagnetic interference and affected the ENF signal estimation. To establish a secondary reliable system, our ongoing work includes developing lightweight estimating method using the ENF from the video recordings and using the proposed technique to achieve a more robust real-time authentication method for smart surveillance.

## Abbreviations

The following abbreviations are used in this manuscript:

ENF	Electrical Network Frequency
FFI	False Frame Injection
STFT	Short Time Fourier Transform
FFT	Fast Fourier Transform
NFFT	Number of FFT bins
SNR	Signal to Noise Ratio
POV	Point of View
QR	Quick Response Code
AC	Alternating Current
CCD	Charge Couple device
CMOS	Complimentary Metal Oxide Semiconductor
FPS	Frames Per Second
HOG	Histogram of Oriented Gradients
PSD	Power Spectral Density
